# Cancers Associated with Human Papillomavirus in American Indian and Alaska Native Populations — United States, 2013–2017

**DOI:** 10.15585/mmwr.mm6937a2

**Published:** 2020-09-18

**Authors:** Stephanie C. Melkonian, S. Jane Henley, Virginia Senkomago, Cheryll C. Thomas, Melissa A. Jim, Andria Apostolou, Mona Saraiya

**Affiliations:** ^1^Division of Cancer Prevention and Control, National Center for Chronic Disease Prevention and Health Promotion, CDC; ^2^Division of Epidemiology and Disease Prevention, STD Program, Indian Health Service, Bethesda, Maryland.

Human papillomavirus (HPV) causes most cervical cancers and some cancers of the penis, vulva, vagina, oropharynx, and anus. Cervical precancers can be detected through screening. HPV vaccination with the 9-valent HPV vaccine (9vHPV) can prevent approximately 92% of HPV-attributable cancers (*1*).* Previous studies have shown lower incidence of HPV-associated cancers in non-Hispanic American Indian and Alaska Native (AI/AN) populations compared with other racial subgroups (*2*); however, these rates might have been underestimated as a result of racial misclassification. Previous studies have shown that cancer registry data corrected for racial misclassification resulted in more accurate cancer incidence estimates for AI/AN populations (*3*,*4*). In addition, regional variations in cancer incidence among AI/AN populations suggest that nationally aggregated data might not adequately describe cancer outcomes within these populations (*5*). These variations might, in part, result from geographic disparities in the use of health services, such as cancer screening or vaccination (*6*). CDC analyzed data for 2013–2017 from central cancer registries linked with the Indian Health Service (IHS) patient registration database to assess the incidence of HPV-associated cancers and to estimate the number of cancers caused by HPV among AI/AN populations overall and by region. During 2013–2017, an estimated 1,030 HPV-associated cancers were reported in AI/AN populations. Of these cancers, 740 (72%) were determined to be attributable to HPV types targeted by 9vHPV; the majority were cervical cancers in females and oropharyngeal cancers in males. These data can help identify regions where AI/AN populations have disproportionately high rates of HPV-associated cancers and inform targeted regional vaccination and screening programs in AI/AN communities.

CDC analyzed cancer incidence data from the United States Cancer Statistics American Indian and Alaska Native Incidence Analytic Database (USCS AIAD), which includes data from central cancer registries that have been linked with the Indian Health Service (IHS) patient registration database (*4*). These methods have been shown to improve the accuracy of estimates of cancer incidence in AI/AN populations^†^ (*3*).

Analyses were restricted to IHS purchased/referred care delivery area (PRCDA) counties, as defined in the October 10, 2017, Federal Register (82 FR 47004). These counties contain or are adjacent to federally recognized tribal lands and have higher proportions of AI/AN residents than do non-PRCDA counties. Data linkages have been shown to be most accurate in these counties (*5*). AI/AN persons accessing services through IHS are members of federally recognized tribes. Analyses were also limited to non-Hispanic populations because previous studies show that updated bridged intercensal population estimates significantly overestimate AI/AN populations of Hispanic origin (*4*).

Cancers were classified by anatomic site using the *International Classification of Diseases for Oncology, Third Edition*^§^ and were confirmed histologically. HPV-associated cancers were defined as invasive cancers at anatomic sites with cell types in which HPV DNA frequently is found, including carcinomas of the cervix (i.e., squamous cell cancers [SCC], adenocarcinomas, and other carcinomas) and SCC of the vulva, vagina, penis, oropharynx, and anus (including rectal SCC) (*1*).

Cancer incidence was expressed as cases per 100,000 population within PRCDA counties and, using 10 age groups, were directly age-adjusted to the 2000 U.S. standard population. Rates among non-Hispanic AI/AN populations were examined by sex, cancer type, and region. Rates by cancer type were compared with those among non-Hispanic White populations in PRCDA counties. Standardized rate ratios (RRs) were used to determine significant differences in rates (p<0.05). Data were suppressed when fewer than six cases were reported.

HPV status is not routinely collected in cancer registries. Therefore, to estimate the number of HPV-attributable cases, the number of HPV-associated cancers was multiplied by the percentage of each cancer type attributable to HPV, based on previous genotyping studies (*3*). Consistent with previous studies, rectal squamous cell carcinoma was not included in the genotyping study, and the HPV-attributable percentage for anal squamous cell carcinoma, a biologically similar tumor, was used (*7*).

For this analysis, PRCDA counties were grouped into six regions: Alaska, East, Northern Plains, Pacific Coast, Southern Plains, and Southwest (Figure). Cervical cancer was the most common HPV-associated cancer in AI/AN females (Table 1) in each region, and rates were significantly higher among AI/AN females than among White females, overall. Cervical cancers accounted for 57% (Northern Plains and the East) to 73% (Southwest) of HPV-associated cancers in AI/AN women. The highest rates of cervical cancer occurred in the Southern Plains (13.8 per 100,000), the lowest occurred in the East (6.5 per 100,000). Rates of other HPV-associated cancers in AI/AN females ranged from 0.7 to 2.6 per 100,000 for cancers of the anus, 0.4 to 3.1 for cancers of the oropharynx, and 0.8 to 3.6 for cancers of the vulva.

**FIGURE:**
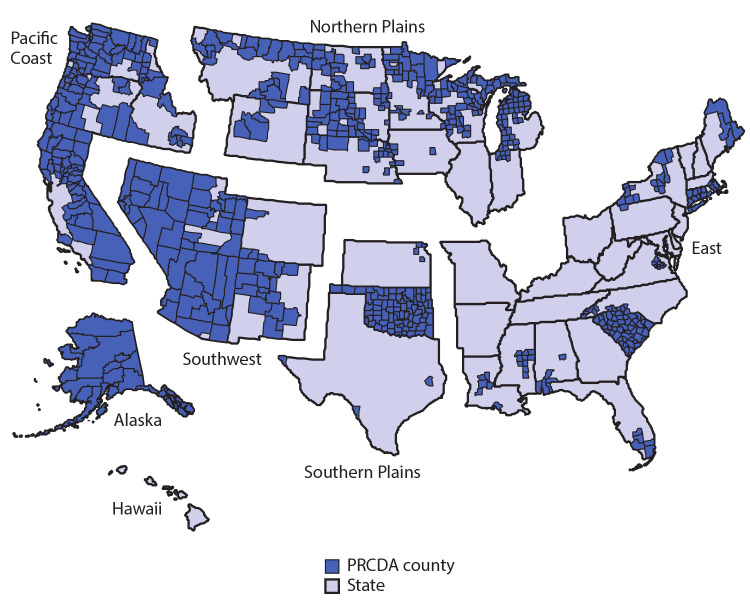
Indian Health Service (IHS) Purchased /Referred Care Delivery Area (PRCDA)* counties, by region — United States, 2013–2017 **Abbreviation**: AI/AN = American Indian and Alaska Native. * PRCDA consist of counties that contain federally recognized tribal lands or are adjacent to tribal lands. Race classification for the AI/AN population is more accurate in these counties. States that have at least one PRCDA-designated county, by region and percentage of total AI/AN population residing in PRCDA counties, include Alaska (100%) (Alaska), Pacific Coast (60.3%) (California, Idaho, Oregon, and Washington), Southwest (83.9%) (Arizona, Colorado, Nevada, New Mexico, and Utah), Northern Plains (54.3%) (Indiana, Iowa, Michigan, Minnesota, Montana, Nebraska, North Dakota, South Dakota, Wisconsin, and Wyoming), Southern Plains (56.7%) (Kansas, Oklahoma, and Texas), and East (16.8%) (Alabama, Connecticut, Florida, Louisiana, Maine, Massachusetts, Mississippi, New York, North Carolina, Pennsylvania, Rhode Island, South Carolina, and Virginia). In the United States, 53.3% of the AI/AN population reside in PRCDA counties.

**TABLE 1:** Incidence* and percent distribution of human papillomavirus (HPV)-associated cancers,^†^ by sex, cancer type, region, and race/ethnicity^§^ — Indian Health Service (IHS) Purchased/Referred Care Delivery Area (PRCDA) counties,^¶^ United States, 2013–2017

Characteristic	AI/AN, rate (%)						All regions combined		
	Northern Plains	Alaska	Southern Plains	Pacific Coast	East	Southwest	AI/AN, rate (%)	White, non-Hispanic rate (%)	RR
**Sex, cancer type**									
**Female**									
All HPV-associated cancers	20.0 (100)	21.6 (100)	21.1 (100)	18.5 (100)	11.1 (100)	8.9 (100)	15.9 (100)	13.7 (100)	1.16**
Cervix	11.2 (57)	12.8 (59)	13.8 (65)	12.6 (63)	6.5 (57)	6.6 (73)	10.3 (63)	6.5 (39)	1.58**
Vagina	—^††^	—	—	—	—	—	0.4 (2)	0.4 (3)	1.11
Vulva	3.6 (16)	3.1 (12)	3.3 (16)	1.1 (8)	1.9 (17)	0.8 (8)	2.0 (13)	2.2 (18)	0.93
Oropharynx	2.3 (11)	3.1 (14)	1.8 (9)	2.1 (14)	—	0.4 (5)	1.5 (10)	1.9 (16)	0.80
Anus	2.6 (13)	2.0 (12)	1.7 (9)	2.5 (15)	—	0.7 (9)	1.7 (11)	2.7 (23)	0.61**
**Male**									
All HPV-associated cancers	10.6 (100)	11.4 (100)	14.9 (100)	12.7 (100)	10.0 (100)	4.1 (100)	10.2 (100)	11.8 (100)	0.86**
Oropharynx	9.0 (86)	6.3 (67)	12.2 (83)	10.3 (81)	8.6 (84)	3.3 (78)	8.2 (82)	9.7 (83)	0.84**
Anus	—	—	1.7 (10)	1.3 (11)	—	—	1.1 (11)	1.4 (11)	0.78
Penis	—	—	1.1 (7)	1.1 (7)	—	0.5 (13)	0.9 (8)	0.7 (6)	1.26

**Abbreviations:** AI/AN = American Indians and Alaska Natives; ICD-O-3 = *International Classification of Diseases for Oncology, Third Edition*; RR = rate ratio.

* Cases per 100,000 persons; age-adjusted to the 2000 U.S. standard population.

^†^ HPV-associated cancers were defined as invasive cancers at anatomic sites with cell types in which HPV DNA frequently is found. All cancers were histologically confirmed. Cervical cancers (ICD-O-3 site codes C53.0–C53.9) are limited to carcinomas (ICD-O-3 histology codes 8010–8671, 8940–8941). Vaginal (ICD-O-3 site code C52.9), vulvar (ICD-O-3 site codes C51.0–C51.9), penile (ICD-O-3 site codes C60.0–60.9), anal (ICD-O-3 site codes C20.9, C21.0–C21.9) and oropharyngeal cancers are limited to squamous cell carcinomas (ICD-O-3 histology codes 8050–8084, 8120–8131).

^§^ AI/AN race was reported by cancer registries or identified through linkage with the IHS patient registration database. To minimize racial/ethnic misclassification, analyses were restricted to AI/AN of non-Hispanic origin.

^¶^ Compiled from data for 2013–2017 from 50 states and the District of Columbia in cancer registries that met data quality criteria and linked with the IHS patient registration database; based on patients who resided in a PRCDA-designated county. States that have at least one PRCDA-designated county, by region and percentage of total AI/AN population residing in PRCDA counties, include Alaska (100%) (Alaska), Pacific Coast (60.3%) (California, Idaho, Oregon, and Washington), Southwest (83.9%) (Arizona, Colorado, Nevada, New Mexico, and Utah), Northern Plains (54.3%) (Indiana, Iowa, Michigan, Minnesota, Montana, Nebraska, North Dakota, South Dakota, Wisconsin, and Wyoming), Southern Plains (56.7%) (Kansas, Oklahoma, and Texas), and East (16.8%) (Alabama, Connecticut, Florida, Louisiana, Maine, Massachusetts, Mississippi, New York, North Carolina, Pennsylvania, Rhode Island, South Carolina, and Virginia). In the United States, 53.3% of the AI/AN population reside in PRCDA counties.

** For all regions combined, the rate among AI/AN was significantly (p<0.05) different from the rate among non-Hispanic Whites.

^††^ Dash indicates that data were suppressed when fewer than six cases were reported.

In AI/AN males, rates of HPV-associated cancers ranged from 10.0 (East) to 14.9 per 100,000 (Southern Plains) (Table 1). Oropharyngeal cancers were the most common cancers among AI/AN males across all regions, accounting for 67% (Alaska) to 86% (Northern Plains) of all HPV-associated cancers. Rates of oropharyngeal cancer were the highest in the Southern Plains (12.2 per 100,000) and lowest in the Southwest (3.3 per 100,000). Rates of other HPV-associated cancers in AI/AN males ranged from 0.5 to 1.7 per 100,000 for cancers of the penis and anus.

For all regions combined, rates of all HPV-associated cancers were higher among AI/AN females than among White females (RR = 1.16) and lower among AI/AN males than among White males (RR = 0.86) (Table 1). Among AI/AN females, 63% of HPV-associated cancers were cervical cancer, compared with 39% in White females. Rates of cervical cancer also were higher among AI/AN females than among White females (RR = 1.58). Rates of cancers of the anus were lower among AI/AN females than among White females (RR = 0.61). In AI/AN males, cancers of the oropharynx represented 82% of HPV-associated cancers, compared with 83% in White males. Rates of oropharyngeal cancers were lower in AI/AN males than in White males (RR = 0.84).

During 2013–2017, among the estimated 500 cancers in AI/AN females that could have been prevented by 9vHPV, 330 were cervical cancers (Table 2). Among AI/AN males, a majority of the estimated 240 cancers that could have been prevented by 9vHPV were cancers of the oropharynx. The largest number of potentially vaccine-preventable cancers in AI/AN occurred among those in the Pacific Coast (180) and Southern Plains (230).

**TABLE 2:** Estimated number of human papillomavirus (HPV)–attributable cancers,* by sex, cancer type,^†^ region, and HPV type,^§^ among American Indians and Alaska Natives^¶^ — Indian Health Service (IHS) Purchased/Referred Care Delivery Area (PRCDA)** counties, United States, 2013–2017

Characteristic	Estimated no.		
	9vHPV-targeted	Other HPV	HPV-negative
**All HPV-associated cancers**	**740**	**90**	**200**
Sex			
Female	500	50	100
Male	240	40	100
Cancer type			
Cervix	330	40	40
Vagina	10	<10	<10
Vulva	50	10	30
Oropharynx	230	40	110
Anus	100	<10	10
Penis	20	<10	10
Region			
Northern Plains	130	20	40
Alaska	60	10	20
Southern Plains	210	30	60
Pacific Coast	180	20	50
East	50	10	10
Southwest	110	10	30

**Abbreviations:** 9vHPV = 9-valent HPV vaccine; ICD-O-3 = *International Classification of Diseases for Oncology, Third Edition*.

* HPV-attributable cancers are cancers that are probably caused by HPV (https://academic.oup.com/jnci/article/107/6/djv086/872092). Estimates for attributable fraction were based on studies that used population-based data from cancer tissue studies to estimate the percentage of those cancers probably caused by HPV.

^†^ HPV-associated cancers were defined as invasive cancers at anatomic sites with cell types in which HPV DNA frequently is found. All cancers were histologically confirmed. Cervical cancers (ICD-O-3 site codes C53.0–C53.9) are limited to carcinomas (ICD-O-3 histology codes 8010–8671, 8940–8941). Vaginal (ICD-O-3 site code C52.9), vulvar (ICD-O-3 site codes C51.0–C51.9), penile (ICD-O-3 site codes C60.0–60.9), anal (ICD-O-3 site codes C20.9, C21.0–C21.9), and oropharyngeal (ICD-O-3 site codes C01.9, C02.4, C02.8, C05.1, C05.2, C09.0, C09.1, C09.8, C09.9, C10.0, C10.1, C10.2, C10.3, C10.4, C10.8, C10.9, C14.0, C14.2 and C14.8) cancer sites are limited to squamous cell carcinomas (ICD-O-3 histology codes 8050–8084, 8120–8131).

^§^ “9vHPV-targeted” includes oncogenic HPV types 16, 18, 31, 33, 45, 52, and 58. “Other HPV” includes other oncogenic HPV types. “HPV-negative” cancers are those that occur at anatomic sites in which HPV-associated cancers are often found, but HPV DNA was not detected. The estimated number of HPV-attributable cancers was calculated by multiplying the number of HPV-associated cancer cases by the percentage of each cancer type attributable to HPV, grouped as types targeted by 9vHPV and other HPV types. HPV-negative estimates were the difference of the total count and the HPV-attributable estimates. Estimates were rounded to the nearest 10; estimates <10 are not displayed.

^¶^ AI/AN race was reported by cancer registries or identified through linkage with the IHS patient registration database. To minimize racial/ethnic misclassification, analyses were restricted to AI/AN of non-Hispanic origin.

** Compiled from data for 2013–2017 from 50 states and the District of Columbia in cancer registries that met data quality criteria and linked with the IHS patient registration database; based on patients who resided in a PRCDA-designated county. States that have at least one PRCDA-designated county, by region and percentage of total AI/AN population residing in PRCDA counties, include Alaska (100%) (Alaska), Pacific Coast (60.3%) (California, Idaho, Oregon, and Washington), Southwest (83.9%) (Arizona, Colorado, Nevada, New Mexico, and Utah), Northern Plains (54.3%) (Indiana, Iowa, Michigan, Minnesota, Montana, Nebraska, North Dakota, South Dakota, Wisconsin, and Wyoming), Southern Plains (56.7%) (Kansas, Oklahoma, and Texas), and East (16.8%) (Alabama, Connecticut, Florida, Louisiana, Maine, Massachusetts, Mississippi, New York, North Carolina, Pennsylvania, Rhode Island, South Carolina, and Virginia). In the United States, 53.3% of the AI/AN population reside in PRCDA counties.

## Discussion

Incidence of HPV-associated cancers in AI/AN populations varied by geographic region and sex. Overall, rates of HPV-associated cancers were higher in AI/AN females, but lower in AI/AN males when compared with rates in the non-Hispanic White population. Cervical cancer and oropharyngeal cancers accounted for the highest incidences, compared with other HPV-associated cancers among AI/AN females and males, respectively.

HPV vaccination is an important element of primary cancer prevention (*8*) and recommended for prevention of all cancer types associated with HPV, including cervical and oropharyngeal cancers.^¶^ The Advisory Committee on Immunization Practices recommends routine HPV vaccination at age 11–12 years and catch-up HPV vaccination for all adults through age 26 years.** The *Healthy People 2020* target is for 80% of teens aged 13–15 years to receive 2 or 3 doses of HPV vaccine.^ ††^ In 2018, approximately 85.1% of IHS adolescent patients aged 13–17 years had received at least their first dose of HPV vaccine, 73.3% had received 2 doses, and 48.4% had received 3 doses.^§§^ First dose HPV vaccination estimates from the National Immunization Survey-Teen are approximately 70% for AI/AN teens, and up-to-date coverage is estimated to be approximately 57.3%.^¶¶^ Despite the high rates of first dose vaccination, HPV vaccination still lags behind coverage for other vaccines administered in the same age range, suggesting that local and culturally tailored interventions might increase coverage (*9*).

In addition to HPV vaccination, screening is an important strategy to prevent cervical cancer, the only HPV-associated cancer that has routine screening recommendations. In 2017, only 54.8% of AI/AN women had been screened according to current cervical cancer screening recommendations, despite the *Healthy People 2020* target of 95% (*10*). Federal programs such as CDC’s National Breast and Cervical Cancer Early Detection Program provide access to cervical cancer screening and diagnostic services to underserved women.*** Partnerships also have been established with tribal programs, states, and other organizations to increase outreach and education for AI/AN women. The current coronavirus disease 2019 (COVID-19) pandemic is potentially disrupting recommended screening and prevention services in underserved populations. Future studies can evaluate the effect of the COVID-19 pandemic on receipt of preventive health services in Indian country.

The findings in this report are subject to at least three limitations. First, population-based cancer registries do not routinely collect or report information on HPV genotype status in cancer registries; therefore, HPV-attributable cancers can only be estimated. Second, this report only includes data for members of federally recognized tribes and those who have accessed services through IHS. Rates might differ for AI/AN populations not included in this report. Finally, although the exclusion of Hispanic AI/AN persons from the analyses reduced the overall AI/AN incidence by less than 5% (*4*), this exclusion might disproportionally affect rates in some states and regions.

Data from the central cancer registries can be used to monitor the long-term effect of HPV vaccination and current cancer screening strategies for AI/AN populations. Understanding the regional variation of HPV-associated cancers can aid in the development of targeted and culturally appropriate interventions to address disparities in AI/AN populations.

**Summary**

**What is already known about this topic?**

Human papillomavirus (HPV) causes nearly all cervical cancers and some cancers of the vagina, vulva, penis, anus, and oropharynx. Racial misclassification of American Indian and Alaska Native (AI/AN) populations in cancer registry data results in cancer incidence underestimates.

**What is added by this report?**

In data from central cancer registries linked with Indian Health Service patient information, 740 (72%) of 1,030 HPV-associated cancers among AI/AN were estimated to be types targeted by 9-valent HPV vaccine. Oropharyngeal cancers were the most common HPV-associated cancers among AI/AN males, and cervical cancers were the most common among AI/AN females.

**What are the implications for public health practice?**

Surveillance for HPV-associated cancers by region can inform local HPV vaccination and cervical cancer screening efforts targeting AI/AN communities.

## Footnotes

* Percentage of each cancer type attributable to HPV based on genotyping studies*.* The denominator is attributable to any HPV; the numerator is attributable to oncogenic HPV types 16, 18, 31, 33, 45, 52, and 58, for both sexes.

† Also available via the data visualization website https://gis.cdc.gov/Cancer/USCS/DataViz.html under Special Topics.

§ http://www.iacr.com.fr/index.php?option=com_content&view=category&layout=blog&id=100&Itemid=577.

¶ https://www.cdc.gov/cancer/hpv/basic_info/.

** https://www.cdc.gov/vaccines/vpd/hpv/hcp/recommendations.html.

†† IID-114 and IID-11.5; https://www.healthypeople.gov/2020/topics-objectives/topic/immunization-and-infectious-diseases/objectives.

§§ https://www.ihs.gov/sites/epi/themes/responsive2017/display_objects/documents/vaccine/reports/FY18_4thQuarter.pdf. IHS does not currently report “up to date” according to those with ≥3 doses, and those with 2 doses when the first HPV vaccine dose was initiated before age 15 years.

¶¶ https://stacks.cdc.gov/view/cdc/80676.

*** https://www.cdc.gov/cancer/nbccedp/index.htm.
